# Geschulte Schlaganfall-Helferinnen und Schlaganfall-Helfer: Ehrenamtliches Versorgungsmodell in der ambulanten Schlaganfallnachsorge

**DOI:** 10.1007/s00391-020-01816-0

**Published:** 2020-11-24

**Authors:** Kerstin Bilda, Stefan Stricker

**Affiliations:** 1 Hochschule für Gesundheit, Gesundheitscampus 6–8, 44801 Bochum, Deutschland; 2 Integrierte Versorgung, Stiftung Deutsche Schlaganfall-Hilfe, Schulstr. 22, 33311 Gütersloh, Deutschland

**Keywords:** Ambulante Gesundheitsdienste, Nachsorge, Ehrenamtliche, Schulung, Gesundheitsbezogene Lebensqualität, Outpatient health services, Aftercare, Voluntary workers, Education, Health-related quality of life

## Abstract

Die Rehabilitation von Menschen mit einem Schlaganfall ist ein langjähriger Prozess, in dem Betroffene, ihre Familien und Freunde auf soziale Unterstützung und Hilfe angewiesen sind. Daher stellt die individuelle Förderung der vorhandenen Ressourcen und der Selbstständigkeit der Betroffenen eine wichtige Aufgabe dar. Die Hochschule für Gesundheit Bochum und die Stiftung Deutsche Schlaganfall-Hilfe haben das ehrenamtsunterstützte Modell „Geschulte Schlaganfall-Helferinnen und Schlaganfall-Helfer – ein Partnerschaftliches Modell für die wohnortnahe Versorgung (GeSa)“ entwickelt, das Schlaganfall-Helfer*innen als Expert*innen für eine individuelle wohnortnahe Unterstützung von Schlaganfall-Patient*innen und ihre Angehörige schult. Die Schlaganfall-Helfer*innen bieten eine patient*innenorientierte Versorgung und individuelle Hilfestellung im Alltag an und leisten somit einen Beitrag zur Verbesserung der gesundheitsbezogenen Lebensqualität von Betroffenen und deren Familien. Es wurde ein Ausbildungscurriculum entwickelt, sowie in einer Schulung mit 21 Schlaganfall-Helferer*innen erfolgreich erprobt und evaluiert. Bis zum Sommer 2020 sind an 16 Standorten in 37 Schulungen über 480 Schlaganfall-Helfer*innen ausgebildet worden. Die bisherigen Erfahrungen mit dem Projekt sind durchgehend positiv, was durch 2 wissenschaftliche Arbeiten bestätigt wird. Als wichtigster Erfolgsfaktor für die Nachhaltigkeit des Projekts hat sich die bereits im Modellprojekt umgesetzte Einbindung von Kooperationspartner*innen für die Koordination der Ehrenamtlichen bewährt. Im Beitrag werden die Ergebnisse der Pilotstudie sowie der standardisierte Prozess zur Implementierung eines regionalen Helfer*innen-Projektes dargestellt und die Erfahrungen mit dem bisherigen Projektverlauf beschrieben.

## Abstract (en)

The rehabilitation of people after suffering a stroke is a long-term process, in which patients, their families and friends are reliant on social support and assistance. Therefore, the individual promotion of available resources and autonomy of those affected represents an important task. The College for Health in Bochum and the German Stroke Help Foundation have developed the volunteer-supported model “Trained stroke helpers—a partnership-based model for local care (GeSa)” that educates stroke helpers as experts for individual local support of stroke patients and their relatives. The stroke helpers offer a patient-oriented care and individual assistance in the daily routine and therefore make a contribution to improvement of the health-related quality of life of those affected and their relatives. A training curriculum was developed and successfully tested and evaluated in a course with 21 stroke helpers. Up to summer 2020 a total of 480 stroke helpers have been trained at 16 locations in 37 courses. The experiences with the project have so far been consistently positive, which was confirmed by two scientific studies. The inclusion of cooperation partners for the coordination of the volunteers, which was already implemented in the model project, has been shown to be the most important success factor for the sustainability of the project. This article presents the results of the pilot study and the standardized process on implementation of a regional stroke helper project and describes the experiences with the previous course of the project.

## Einleitung

Der Schlaganfall ist eine Herz-Kreislauf-Erkrankung, die ca. 270.000 Menschen jedes Jahr erleiden. Die Überlebensrate nach einem Schlaganfall ist aufgrund der sehr guten medizinischen Versorgung in der Akutphase, speziell durch die „stroke units“, sehr hoch [9, 14]. Allerdings müssen sich Menschen, die einen Schlaganfall überleben, zumeist mit verschiedenen Funktionseinschränkungen auseinandersetzen. Diese Einschränkungen haben erheblichen Einfluss auf die Lebensqualität und die damit einhergehende soziale Teilhabe im Alltag und wirken sich auch körperlich und emotional auf das Leben der Angehörigen aus. Eine selbstbestimmte Teilhabe an der Gesellschaft ist Schlaganfallbetroffenen oft nicht mehr möglich. Die wohnortnahe Versorgung ist oft nicht ausreichend, um psychische Gesundheit, emotionale Stabilität und soziale Teilhabe in der ambulanten Nachsorge von Schlaganfallpatient*innen zu gewährleisten.

Vor diesem Hintergrund haben die Hochschule für Gesundheit und die Stiftung Deutsche Schlaganfall-Hilfe ein wohnortnahes ehrenamtliches Versorgungsmodell für die Langzeitnachsorge bei Schlaganfall entwickelt, erprobt und wissenschaftlich ausgewertet. Das Modellprojekt „Geschulte Schlaganfall-Helferinnen und Schlaganfall-Helfer – ein Partnerschaftliches Modell für die wohnortnahe Versorgung (GeSa)“ (Laufzeit 2013–2015) wurde über das Ministerium für Gesundheit, Emanzipation, Pflege und Alter des Landes Nordrhein-Westfalen (MGEPA NRW) und den Europäischen Fonds für regionale Entwicklung finanziell gefördert. Das Ziel des Projekts war es, ein ehrenamtsunterstütztes Versorgungsmodell für die ambulante Versorgung von Patient*innen und Angehörigen nach Schlaganfall zu entwickeln und wissenschaftlich im Hinblick auf die Wirksamkeit zu evaluieren. Um die gesundheitsbezogene Lebensqualität von Betroffenen und deren Angehörigen nachhaltig zu verbessern, wurden 21 Schlaganfall-Helfer*innen in einer Schulung dazu befähigt, individuelle fachliche und emotionale Unterstützung in der ambulanten Langzeitnachsorge zu übernehmen. Das Forschungsprojekt konnte nachweisen, dass die geschulten Helfer*innen wirksame Unterstützung und Beratung in der häuslichen Versorgung nach Schlaganfall geleistet haben [2].

Strukturell ist die Funktion der ehrenamtlich tätigen Schlaganfall-Helfer*innen komplementär zu professionellen Schlaganfall-Lots*innen [4] angelegt, die in enger Zusammenarbeit als kollegiales Team organisiert sind. Darüber hinaus sind die ehrenamtlichen Schlaganfall-Helfer*innen lediglich als ergänzendes Angebot in der Schlaganfallversorgung verortet. Ein Ersatz für professionelle Leistungsangebote (z. B. Pflegedienste) ist nicht vorgesehen, stattdessen können Schlaganfall-Helfer*innen den professionellen Leistungserbringer*innen eine unterstützende Hilfe für ihre Tätigkeit sein [1].

Mit dem Ende des Modellprojekts hat die Stiftung Deutsche Schlaganfall-Hilfe ab 2015 begonnen, das Projekt bundesweit zu vervielfältigen. Dafür wurden standardisierte Umsetzungsprozesse sowie verschiedene organisatorische und strukturelle Rahmenbedingungen für die praktische Weiterentwicklung definiert und weiterentwickelt.

## Ehrenamtliches Versorgungsmodell

Die primäre Aufgabe der Schlaganfall-Helfer*innen besteht darin, die Betroffenen und Angehörigen individuell in ihrem Lebensalltag zu begleiten und zu unterstützen. Da die wohnortnahen Schlaganfall-Helfer*innen mit den regionalen Versorgungsstrukturen sehr vertraut sind, können sie schnell und effektiv bei Herausforderungen im Alltag helfen (z. B. wohnortnaher Zugang zu Kontaktstellen, Ausfüllen eines Formulars, Führen eines Telefonats). Als Vertrauensperson und kontinuierliche Begleitung können sie die Selbstständigkeit und Krankheitsbewältigung fördern sowie der sozialen Isolation vorbeugen.

Des Weiteren tragen Schlaganfall-Helfer*innen dazu bei, „Fehlallokationen“ in der Gesundheitsversorgung frühzeitig zu erkennen und für Abhilfe zu sorgen. Beispielsweise kann ein falscher Rollstuhl langfristig weitere Kosten verursachen, weil er eine Fehlhaltung herbeiführt, die Rückenbeschwerden auslösen kann. Eine besondere Rolle spielen in diesem Kontext falsch behandelte psychische Folgen eines Schlaganfalls (Depressionen). Werden diese rechtzeitig erkannt und behandelt, können erhebliche Kostensteigerungen in der Schlaganfallversorgung vermieden werden.

## Forschungsleitende Fragestellungen

Folgende Fragestellungen wurden im Rahmen der wissenschaftlichen Analyse untersucht:

1. Können geschulte Schlaganfall-Helfer*innen einen wirksamen Beitrag zur Verbesserung der Lebensqualität und der sozialen Teilhabe von Patient*innen mit Schlaganfall in der häuslichen Versorgung leisten?
2. Welche Faktoren tragen zur Entlastung der Angehörigen bzw. anderer unterstützender Personen bei?

## Entwicklung der Ausbildungsinhalte

In einer qualitativen Vorstudie wurden 20 Fokusinterviews mit Expert*innen aller Segmente der Versorgungskette geführt, um u. a. Lücken im Versorgungssystem zu entdecken und diese bei der Modulentwicklung zu berücksichtigen. Zu den Expert*innen gehörten Schlaganfallbetroffene, Angehörige, Schlaganfall-Lots*innen, Therapeut*innen, Rechtsberater*innen, Ärzte/Ärztinnen von Rehakliniken wie auch Stroke units, die ambulante Pflege, Selbsthilfegruppen und Gesundheitswissenschaftler*innen. Die Kernaussagen und fachlichen Schwerpunkte wurden in die Ausbildungsmodule integriert.

Auf der Grundlage dieser Analysen wurde ein Curriculum mit 10 Modulen entwickelt, das fachspezifisch für die professionelle Beratung und Begleitung von Menschen mit Schlaganfall qualifiziert. Die Themen umfassten folgende Inhalte:

- medizinische Grundlagen des Schlaganfalls,
- Therapie der Funktionsstörungen bei Schlaganfall (Motorik, Kognition, Sprache),
- Sozialrecht,
- Gesprächsführung und Beratung,
- Selbsthilfe.

## Auswahl und Vorbereitung der Referent*innen

Bei der Auswahl externer Referent*innen für die Schulungen wurden gezielt Personen ausgewählt, die bereits über jahrelange Erfahrungen mit der Schulung von Schlaganfallbetroffenen und deren Angehörigen verfügen. Aufgrund dieser umfassenden Erfahrungen kannten die Referent*innen die spezifischen Anforderungen an Schulungsmaterial und didaktisches Vorgehen im Kontext der Schulung von Schlaganfallbetroffenen. Dies bezog sich sowohl auf die typischen Fragestellungen und Problemlagen nach einem Schlaganfall als auch auf deren Kenntnisse hinsichtlich der Ausgestaltung der Schulungsmaterialien. Darüber hinaus wussten sie, wie und in welchem Ausmaß Schlaganfallbetroffene im Rahmen einer Schulungsmaßnahme aufgrund ihrer krankheitsbedingten physischen und psychischen Einschränkungen belastbar sind. Die überwiegende Mehrheit der Referent*innen hatte bereits mit der Deutschen Schlaganfall-Stiftung zusammengearbeitet.

Alle Referent*innen waren für die Entwicklung und Auswertung ihrer Schulungsmodule verantwortlich.

## Erste Pilotschulung

**Figure 1:**
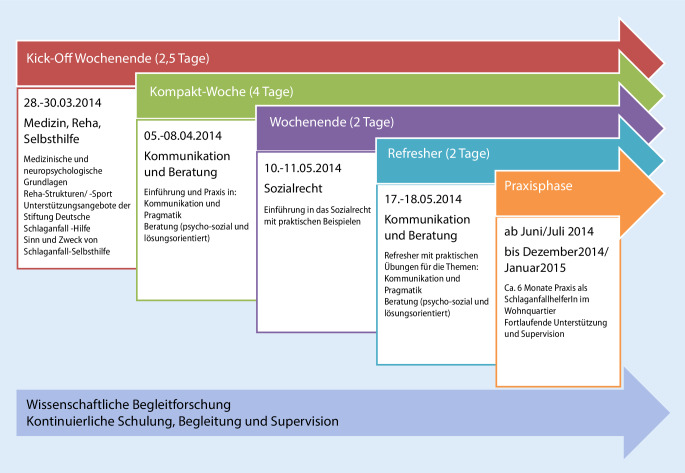

Das Curriculum wurde im Rahmen einer praktischen Ausbildung mit 21 freiwilligen Schlaganfall-Helfer*innen erprobt. Primäre Zielgruppe für die Schlaganfall-Helfer*innen waren sowohl Betroffene mit einer Restsymptomatik als auch im Ehrenamt tätige Nichtbetroffene. Nach der 3‑monatigen Ausbildung wurden 15 geschulte Schlaganfall-Helfer*innen Patient*innen einer Modellregion zugeordnet, in denen sie 6 Monate systematische Unterstützungs- und Beratungsarbeit geleistet haben. In Abb. 1 werden der zeitliche und inhaltliche Verlauf der Pilotschulung dargestellt.

## Evaluation der Praxisphasen

Die Praxisphase der Schlaganfall-Helfer*innen wurde wissenschaftlich begleitet. Nach der im Durchschnitt 6 Monate langen Praxisphase von 15 aktiven ehrenamtlichen Schlaganfall-Helfer*innen wurde der Einsatz mithilfe qualitativer und quantitativer Methoden evaluiert.

### Quantitative Methode

#### Evaluationsinstrumente

Vor Beginn und nach Abschluss des Praxiseinsatzes wurden folgende standardisierte Fragebogen eingesetzt:

- Die häusliche Pflege-Skala wurde 1993 von Gräßel entwickelt. Sie dient der Erfassung der Belastung von pflegenden Angehörigen. Der Fragebogen wird von den Angehörigen selbst ausgefüllt und besteht aus 28 Items [7].
- Der Fragebogen zur sozialen Unterstützung (FSoZu) von Fydrich et al. aus dem Jahr 2007 dient der Erfassung subjektiv wahrgenommener Unterstützung aus dem sozialen Umfeld. Hierbei geht es zum einen um das Gefühl, im Bedarfsfall Unterstützung von anderen Menschen erhalten zu können, und zum anderen um die Einschätzung der eigenen Ressourcen in Bezug auf das soziale Umfeld [5].
- Der Fragebogen zum Gesundheitszustand (SF-36) wurde von Morfeld et al. entwickelt. Genutzt wurde die 2. Auflage aus dem Jahr 2011. Der SF-36 umfasst die Erhebung der gesundheitsbezogenen Lebensqualität von Menschen und kann krankheitsübergreifend eingesetzt werden [12].

Um die Wirksamkeit des Einsatzes hinsichtlich der Qualität der Lebenssituation der betreuten Familien zu messen, wurde für beide Testzeitpunkte und für jedes Testitem der Mittelwert (M) berechnet. Die Berechnung erfolgte durch das Softwarepaket IBM Statistics SPSS (IBM, Ehningen, Deutschland). Weiter wurden die Daten mit dem nichtparametrischen Testverfahren Wilcoxon-Paarvergleichstest auf signifikante Zusammenhänge überprüft.

#### Ergebnisse

Insgesamt haben 8 Angehörige die häusliche Pflege-Skala zu Beginn und zum Ende der Begleitung durch die Schlaganfall-Helfer*innen ausgefüllt. Bei der ersten Erhebung gaben, ausgehend vom Gesamtwert, 89 % der Angehörigen eine mittlere Belastung und 11 % eine hohe Belastung durch die Pflegesituation an. Diese Verteilung ändert sich im Laufe der Begleitung nicht signifikant. Es konnte auch kein signifikanter Unterschied des Gesamtwerts der Belastung zwischen dem ersten und dem zweiten Zeitpunkt festgestellt werden: t(7) = −0,970, *p* = 0,364. Z = −1,192, *p* = 0,233.

Der FSoZu wurde insgesamt von 7 Betroffenen und 8 Angehörigen jeweils zu Beginn und zum Ende des Projekts ausgefüllt. Bei der Erhebung zu Beginn des Projekts wurden die Betroffenen und Angehörigen durch die Schlaganfall-Helfer*innen beim Ausfüllen des Fragebogens unterstützt. Insgesamt zeigte sich ein positiver Trend in der Einschätzung der sozialen Unterstützung zu 2 Messzeitpunkten, der allerdings nicht signifikant war.

Insgesamt haben 8 Schlaganfallbetroffene den SF-36 zu 2 unterschiedlichen Zeitpunkten ausgefüllt. Zunächst zu Beginn der Begleitung durch einen/eine Schlaganfall-Helfer*in und ein zweites Mal zum Ende des Projekts. Die Betroffenen geben zum zweiten Zeitpunkt der Messung über alle Skalen hinweg niedrigere Werte an. Allerdings sind diese Unterschiede zwischen den Messzeitpunkten bei keiner der 8 Subskalen und auch bei keiner der beiden Dimensionen signifikant.

#### Diskussion

Die Stichprobe mit 8 Teilnehmer*innen war sehr klein, somit war eine statistische Analyse und Aussage nur begrenzt möglich.

Aufgrund von personellen Ressourcen im Projekt waren die Durchführungsbedingungen der beiden Testtermine bei allen 3 Fragebogen unterschiedlich. Eine persönliche Befragung war nur zum ersten Messzeitpunkt möglich. Der zweite Messzeitpunkt wurde telefonisch durchgeführt. Alle 3 Fragebogen erfragen stabile und lang andauernde Faktoren als Folgen einer Erkrankung. Die Wirkung einer zeitlich relativ kurzen Begleitung der Schlaganfall-Helfer*innen in individuell sehr unterschiedlichen Phasen der Erkrankungen bei den Schlaganfallbetroffenen lassen sich vermutlich in den eingesetzten standardisierten Fragebogen nicht abbilden.

### Qualitative Methoden

In einem Prä-post-Design wurden insgesamt 35 leitfadengestützte Interviews mit Schlaganfall-Helfer*innen, Betroffenen und Angehörigen vor und nach dem Einsatz der Schlaganfall-Helfer*innen durchgeführt. Die erhobenen Daten wurden qualitativ im Hinblick auf die Wirksamkeit des Einsatzes der Schlaganfall-Helfer*innen ausgewertet. Die Datenauswertung der Interviews erfolgte anhand der computergestützten Analyse qualitativer Daten mit der Software MAXQDA.

#### Ergebnisse der Logbücher

Die Ergebnisse der Logbücher wurden in die Analysen der Wirksamkeit der Begleitung durch die Schlaganfall-Helfer*innen einbezogen. In der Kategorie Reflexion und Wertung war bei allen Helfer*innen eine etwas eingeschränkte Fähigkeit zur Reflexion des eigenen Handels und der eigenen Wertigkeit der Begleitung festzustellen. Je nach Art der Begleitung zeigt sich entweder eine deutliche Unterschätzung der eigenen Leistung (klientenzentriert) oder eine Überschätzung der eigenen Kompetenzen (helferzentriert).

In der Kategorie Begleitung und Nähe-Distanz-Verhalten war das Ziel der Begleitung die Entlassung der Betroffenen in die Selbstständigkeit. Dieses Ziel wird v. a. über eine klientenzentrierte Tätigkeit angestrebt, bei der es darum geht, nicht direkt einzugreifen, sondern den*die Betroffene*n zu selbstgesteuerter Hilfe anzuleiten. Eine solche effektive Haltung scheint mit einem gleichberechtigten Fokus auf Betroffenen und Angehörigen in der Begleitung zu liegen und gleichzeitig mit einem positiven Nähe-Distanz-Verhalten in Verbindung zu stehen.

## Ergebnisse der Interviews mit Helfer*innen, Betroffenen und Angehörigen zur Praxisphase

Eine Besonderheit im Projekt war, dass etwa die Hälfte der Schlaganfall-Helfer*innen (7 von 15) selbst Schlaganfallbetroffene sind. Der Austausch zwischen 2 Betroffenen (Helfer*in und Patient*in) führte zu einer Gegenseitigkeit („Reziprozität“). Durch den Perspektivenwechsel gewinnen begleitete Betroffene Mut und die Erkenntnis, dass es auch andere schaffen. Helfer*innen dienen somit als eine Art Vorbild. Für sie findet ebenfalls eine Stärkung statt, denn sie reflektieren die eigene Entwicklung und Fähigkeit, nun anderen Menschen durch ihre Erfahrungen helfen und diese motivieren zu können. Deshalb entsteht durch diese Reziprozität eine Win-win-Situation, von der beide Seiten profitieren.

In den Interviews stellte sich heraus, dass die gemeinsame Basis „Schlaganfallerfahrung“ eine Vertrauensbasis schafft und somit die Zusammenarbeit beschleunigt und den Zugang zur betroffenen Person erleichtert. Die Schlaganfall-Helfer*innen gaben an, durch die fachliche Schulung gut für ihren Einsatz vorbereitet gewesen zu sein, und bewerteten den Einsatz insgesamt als sehr lohnenswert, da viele wertvolle und sinnstiftende Erfahrungen gesammelt wurden.

### Aufbau und Inhalte der Begleitung

**Figure 2:**
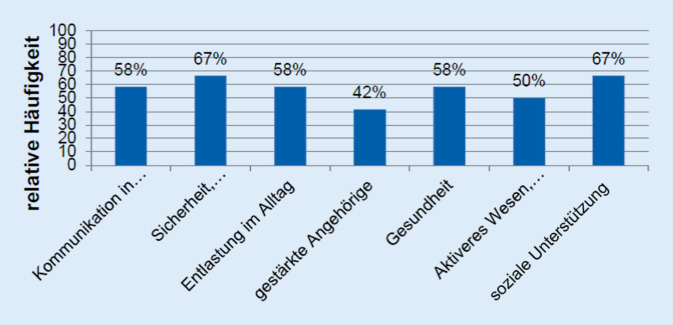

Die organisatorische und inhaltliche Begleitung wurde von den Schlaganfall-Helfer*innen individuell auf die Bedürfnisse der Familien abgestimmt. Die Begleitung fand telefonisch und/oder in persönlichen Treffen statt. Die Treffen dauerten zwischen einer und zwei Stunden. Die zentralen Themen und Aufgaben sowie ihre Wirksamkeit sind in Abb. 2 dargestellt.

Der Einsatz der Schlaganfall-Helfer*innen führte bei den Schlaganfallbetroffenen zu mehr Aktivitäten, zu mehr Selbstvertrauen und somit insgesamt zu einer Steigerung der Lebensqualität. Diese positiven Effekte konnten ebenfalls bei den Angehörigen nachgewiesen werden. Weiter haben Angehörige die verbesserte Kommunikation mit dem/der Partner*in und innerhalb der Familie (Kinder, Enkelkinder) als positiven Effekt durch die individuelle Begleitung angegeben. Insgesamt zeigte sich, dass die besondere Stärke der Schlaganfall-Helfer*innen darin lag, flexibel und offen auf die Diversität der Kontexte bei den Betroffenen und Angehörigen zu reagieren und agieren. Die Begleitung durch geschulte Schlaganfall-Helfer*innen führte zu einem Gefühl von Sicherheit und Orientierung bei den Familien und gleichzeitig zu einer nachhaltigen Optimierung von Strukturen und Prozessen.

## Start der bundesweiten Umsetzung des Projektes

Nach der Beendigung des Modellprojekts im Jahr 2015 wurde seitens der Stiftung Deutsche Schlaganfall-Hilfe die bundesweite Umsetzung des Projekts vorbereitet. Diese Umsetzung erfolgte in 3 Phasen:

- Vorbereitungsphase (2015–2016),
- Erprobungsphase (2016–2019),
- Umsetzungs- und Skalierungsphase (Start in 2020).

In der *Vorbereitungsphase* des Projekts wurden zunächst die organisatorischen und strukturellen Rahmenbedingungen für die künftige bundesweite Umsetzung geschaffen. Dazu gehören die

- Erstellung eines „Manuals zur Organisation einer Schlaganfall-Helfer*innen-Schulung“, das potenziellen regionalen Projektpartnern als Leitfaden zur Umsetzung des Projekts vor Ort dienen soll;
- Einrichtung einer Internetseite, die mehrere Funktionen erfüllen soll (allgemeine Informationsplattform zum Projekt, Service-Bereich mit Download-Angeboten für potenzielle und bestehende Projektpartner*innen, Kurzversion des Manuals);
- systematische Recherche potenzieller Projektpartner*innen anhand verschiedener Kriterien (möglichst breite Streuung in der Fläche, gesicherte Nachhaltigkeit, Erfahrungen im Ehrenamtsmanagement, Reichweite, Synergieeffekte mit anderen Stiftungsprojekten);
- Aufbau einer dauerhaften begleitenden Evaluation der Helfer*innen-Tätigkeiten: sowohl die Wirksamkeit des Helfer*innen-Einsatzes als auch die begleitende Unterstützung der Helfer*innen (Schulungsinhalte, Materialien, etc.) sollen damit laufend hinsichtlich ihrer Qualität überprüft werden;
- Erstellung von Materialien für die Öffentlichkeitsarbeit des Projekts (Projektflyer, Poster, Roll-up, Präsentationen etc.);
- Anerkennung der Schulung im Rahmen der §§45b und c SGB XI, sodass die ehrenamtlichen Schulungsteilnehmer*innen berechtigt sind, ihre Leistungen als „Angebote zur Unterstützung im Alltag“ mit den Pflegekassen abrechnen zu können.^1^

In der *Erprobungsphase* wurde das Projekt an verschiedenen Standorten in Deutschland in die Praxis umgesetzt, um die dabei gewonnenen Erkenntnisse und Erfahrungen für die weitere Projektentwicklung zu nutzen. Bereits im Frühjahr 2016 fand in Gütersloh die erste Schulung von 13 weiteren Schlaganfall-Helfer*innen statt. Noch im gleichen Jahr konnten an 3 weiteren Standorten Schulungen mit insgesamt 49 Schulungsteilnehmer*innen realisiert werden. Parallel dazu hat die Stiftung die Öffentlichkeitsarbeit zu dem Projekt intensiviert, sodass weitere Organisationen und Institutionen darauf aufmerksam gemacht werden konnten. Bis zum 31.07.2020 sind an 16 Standorten in 37 Schulungen über 480 Schlaganfall-Helfer*innen ausgebildet worden. Dabei wurden die zuvor entwickelten Angebote und Services direkt in der Praxis erprobt, evaluiert und im Einzelfall ggf. modifiziert. Teilweise wurden regionalspezifische Besonderheiten berücksichtigt, die sich auch konzeptionell und in der Notation ausgewirkt haben.^2^

**Figure 3:**
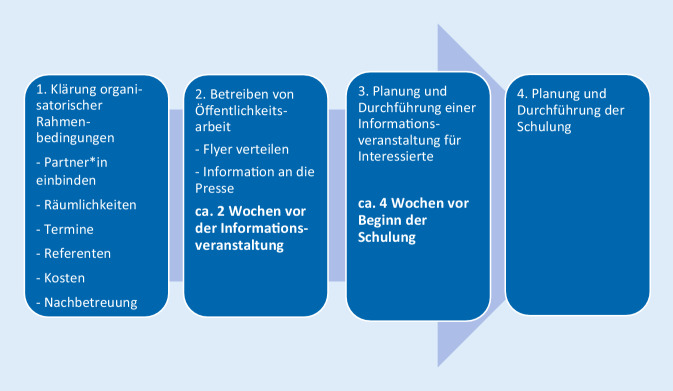

Aufgrund dieser praktischen Erfahrungen konnte ein standardisierter Umsetzungsprozess entwickelt werden, der als Handlungsempfehlung für neu geplante Helfer*innen-Projekte bereits mehrfach erfolgreich verwendet worden ist. In der Praxis hat sich gezeigt, dass vor der Durchführung der eigentlichen Schulung eine öffentliche Informationsveranstaltung für interessierte Personen sinnvoll ist. Um jedoch überhaupt Personen auf die Schulung aufmerksam zu machen, bedarf es gezielter Öffentlichkeitsarbeit. Zu Beginn sollten jedoch zunächst die organisatorischen Rahmenbedingungen geklärt werden. Es empfiehlt sich daher, die in Abb. 3 aufgelisteten 4 Planungsbausteine in chronologischer Reihenfolge abzuarbeiten.

Zur Sicherstellung der Nachhaltigkeit des Schlaganfall-Helfer*innen-Projekts ist es erforderlich, dass die geschulten Schlaganfall-Helfer*innen vor Ort begleitet und betreut werden. In der Praxis der bisherigen Helfer*innen-Projekte hat sich dafür die Einrichtung einer Projektkoordinationsstelle bewährt. Diese Koordinationsstelle kann bei einem/einer Projektpartner*in angebunden sein oder ist bereits organisatorischer Bestandteil der Institution, die die Helfer*innen-Schulung durchführt.

Die Koordinator*innen werden durch ein ausführliches Manual organisatorisch auf ihre Tätigkeit vorbereitet.^3^ Inhaltlich können sie sich durch die Teilnahme an den Schulungen zusätzlich auf die zukünftige Tätigkeit vorbereiten. Darüber hinaus stehen die zuständigen Projektmitarbeiter*innen in der Stiftung jederzeit für weitere Rückfragen zur Verfügung.

Auch der Prozess der Vermittlung der ehrenamtlichen Helfer*innen an Hilfesuchende ist aufgrund der Erfahrungen der Erprobungsphase standardisiert worden. Die Vermittlung der Schlaganfall-Helfer*innen an Hilfesuchende erfolgt über die Projektkoordinationsstelle.

**Figure 4:**
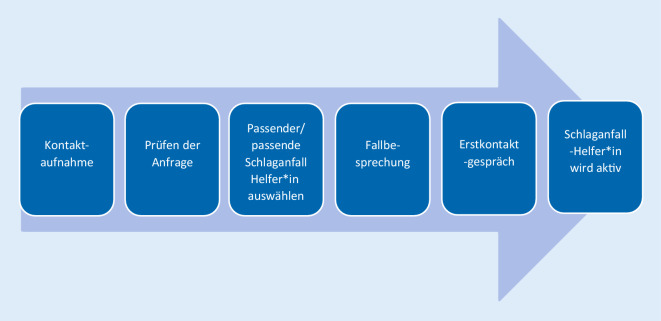

Bei der Kontaktaufnahme prüft die Projektkoordination zunächst, ob die angefragte Hilfe überhaupt durch einen/eine Schlaganfall-Helfer*in erbracht werden kann (Abb. 4). Dazu sollen möglichst detaillierte Informationen abgefragt werden, um eine entsprechende Bewertung vornehmen zu können. Im Idealfall findet sogar ein erstes persönliches Gespräch der Projektkoordination mit dem/der Hilfesuchenden statt.^4^ Danach kann entschieden werden, ob die angefragte Hilfe leistbar ist, oder ob auf andere (professionelle) Hilfen verwiesen werden muss.

Im nächsten Schritt prüft die Projektkoordination, welcher/welche Schlaganfall-Helfer*in für die angefragte Unterstützung infrage kommt. Neben der zeitlichen Verfügbarkeit können weitere Kriterien der Schlaganfall-Helfer*innen wie räumliche Präferenzen, Wünsche an das Tätigkeitsprofil, personenbezogene Aspekte (Geschlecht, Alter etc.) eine Rolle spielen. Als Hilfestellung kann ein eigens dafür entwickelter Matching-Leitfaden verwendet werden.

Nachdem passende Schlaganfall-Helfer*innen gefunden worden sind, bespricht die Projektkoordination den Fall mit ihnen und entwickelt erste Ideen für eine konkrete Hilfe. Im Idealfall führen die Helfenden anschließend gemeinsam den Erstbesuch durch.^5^

Beim Erstgespräch sollten Helfende und Hilfesuchende sich kennenlernen und besprechen, wie die Unterstützung aussehen kann. Dafür steht ein Erstkontaktbogen zur Verfügung.^6^ Danach können die Schlaganfall-Helfer*innen gemäß der Absprache aktiv werden.

In der *Umsetzungs- und Skalierungsphase* wurden gezielt überregional tätige Organisationen über das Projekt informiert und auf eine mögliche Zusammenarbeit angesprochen. Ziel ist es, in den ausgewählten Regionen Schlaganfall-Helfer*innen-Schulungen auf den Weg zu bringen, um vor Ort dauerhaft Helfer*innen-Strukturen aufzubauen. Aufgrund der Einschränkungen durch die Coronapandemie ab Februar 2020 konnten bisher nur erste Gespräche mit den Organisationen geführt werden. Die konkrete Planung von Helfer*innen-Schulungen ist jedoch zunächst verschoben worden.

## Schlaganfall-Helfer*innen in der Praxis

Die bisherigen Erfahrungen mit dem Projekt sind durchgehend positiv, was auch durch 2 wissenschaftliche Arbeiten bestätigt wird [3, 8]. Die Schlaganfall-Helfer*innen werden als „sinnvolle Ergänzung zur Regelversorgung“ beschrieben. Als besonderer Vorteil der ehrenamtlichen Schlaganfall-Helfer*innen wird zudem angeführt, dass die persönliche Betroffenheit von ihnen Angehörige und Betroffene zusätzlich motivieren kann. Außerdem profitieren professionelle Leistungserbringer*innen, da die Helfer*innen ihnen zeitaufwendige Beratungsleistungen abnehmen [8]. In einer weiteren Studie wurden die Faktoren betrachtet, die die Implementierung von Schlaganfall-Helfer*innen vor Ort beeinflussen. Dabei wurde festgestellt, dass v. a. die Schulung die späteren Schlaganfall-Helfer*innen sehr gut auf ihre ehrenamtliche Tätigkeit vorbereitet. Außerdem wurden die organisatorischen Rahmenbedingungen in Form des Vorhandenseins einer Projektkoordination beim Helfer*innen-Projekt vor Ort als positiver Erfolgsfaktor identifiziert [3]. Der letztgenannte Punkt deckt sich auch mit den bisherigen Projekterfahrungen. Alle Helfer*innen-Projekte, bei denen die Koordinationsstelle ihre Tätigkeit eingestellt hat, sind mittelfristig zum Erliegen gekommen. Insofern kann die bereits im Modellprojekt umgesetzte Einbindung von Kooperationspartner*innen für die Koordination der Ehrenamtlichen als einer der wichtigsten Erfolgsfaktoren für die Nachhaltigkeit des Projektes bezeichnet werden.

Im Rahmen einer Befragung der verschiedenen Helfer*innen-Projekte im Mai 2020 durch die Stiftung wurden verschiedene Informationen zur Art der Begleitung eingeholt. Diese erfolgt fast ausschließlich persönlich. In lediglich 5 % der Fälle blieb es bei einer rein telefonischen Beratung. Vermutlich wäre die Zahl der telefonischen Beratungen höher, wenn der Kontakt zwischen Helfer*innen und Hilfesuchenden nicht über die Koordinationsstelle laufen würde. Hier werden bereits erste Anfragen ausgefiltert und ggf. an andere Stellen weitergeleitet. Beim Mentor*innen-Projekt in Schleswig-Holstein erfolgt der Kontakt direkt mit dem/der Mentor*in, sodass die Filterfunktion der Koordinationsstelle durch die Mentor*innen übernommen werden und die Zahl der telefonischen Beratungen höher ist [8].

Die Art der Begleitung ist sehr vielfältig. Im Vordergrund stehen v. a. allgemeine Gesprächssituationen im Sinne einer Small-Talk-Konversation, die in 80 % der Fälle genannt worden sind. Bei den Gesprächen ging es v. a. um alltägliche Dinge. Gerade bei alleinstehenden Betroffenen mit großen Mobilitätseinschränkungen waren die Schlaganfall-Helfer*innen oft die einzigen Menschen, mit denen die Betroffenen im Laufe der Woche gesprochen haben. Mehrere Schlaganfall-Helfer*innen berichten, dass viele Betroffene sich einsam fühlen, aber auch, dass Angehörige froh sind, wenn sie sich außerhalb des familiären Umfelds mit einer Person austauschen können, die nachvollziehen kann, in welcher Lage sich der/die Betroffene oder der/die Angehörige befindet. In diesem Zusammenhang spielte auch sehr oft (67 %) der Aspekt der Motivation der Betroffenen eine große Rolle. Mehrere Schlaganfall-Helfer*innen gaben an, dass regelmäßig ermutigende und aufmunternde Gespräche erforderlich waren. Bei etwa 59 % der Begleitungen wurden gemeinsame Spaziergänge durchgeführt. Diese dienten einerseits dazu, die Mobilität der Betroffenen zu verbessern. Andererseits konnten im Rahmen eines Spaziergangs ungestört Gespräche geführt werden. Mehrere Schlaganfall-Helfer*innen gaben an, dass diese gemeinsamen Spaziergänge für viele Betroffene die einzige Möglichkeit sei, sich außerhalb des häuslichen Umfelds zu bewegen. Darüber hinaus spielt die Informationsvermittlung bei 55 % der Begleitungen eine wichtige Rolle. In 36 % der Fälle erfolgt eine Unterstützung bei der Bearbeitung von schriftlichen Anträgen (z. B. bei der Krankenkasse oder bei behördlichen Anliegen). In 24 % der Fälle haben die Schlaganfall-Helfer*innen auch bei der Kommunikation mit Externen (Pflegedienst, Ärzt*in, Therapeut*in etc.) unterstützt oder zu externen Terminen begleitet (u. a. auch Einkaufen; 14 %).

Bei der Zahl der Termine pro Begleitung gibt es einen großen Spielraum. Während die meisten Einsätze mit 2 bis 4 Terminen auskommen (70 %), kann es in Einzelfällen im Laufe von 6 Monaten sogar zu mehr als 10 Terminen (24 %) oder auch nur zu einem Termin (6 %) kommen. Die Dauer der einzelnen Termine bewegt sich im Schnitt zwischen 1 und 2 h.

## Diskussion

Bei der Tätigkeit der Schlaganfall-Helfer*innen handelt es sich um eine Form des freiwilligen Engagements, die in mehrfacher Hinsicht die Kriterien eines „modernen Ehrenamts“ [15] oder eines „neuen Ehrenamts“ [11] erfüllt. Die Schlaganfall-Helfer*innen können flexibel selbst bestimmen, in welchem zeitlichen Umfang sie aktiv sind und welche Form von Begleitung sie ausüben. Außerdem bietet die Tätigkeit die Möglichkeit einer Selbstentfaltung und Selbstverwirklichung, verknüpft mit einem Engagement für andere. In diesem Zusammenhang ist eine Win-win-Situation für Helfer*innen und Hilfesuchende vorhanden, insbesondere dann, wenn Helfende gleichsam auch Betroffene sind und durch die Tätigkeit zusätzliches Selbstvertrauen gewinnen. Des Weiteren können die Schlaganfall-Helfer*innen eigene Ideen und Vorstellungen verwirklichen.

Für die jeweiligen Partnerorganisationen wird die Beteiligung an dem Projekt zusätzlich attraktiv, weil sie die Möglichkeit haben, die Tätigkeiten der Schlaganfall-Helfer*innen im Rahmen der §§45b und c SGB XI als „Angebote zur Unterstützung im Alltag“ mit den Pflegekassen abrechnen zu können. Darüber hinaus erzeugt das Projekt fast immer sehr viel öffentliche Aufmerksamkeit. Durch die von der Stiftung entwickelten Leitfäden, Mustertexte und Formulare ist es zudem für die Organisationen relativ einfach möglich, das Projekt vor Ort umzusetzen.

In der Praxis hat sich zusätzlich ein positiver Effekt für die Selbsthilfestrukturen vor Ort herauskristallisiert. Die Information über Selbsthilfeangebote durch die Helfer*innen machen Hilfesuchende auf die Schlaganfallselbsthilfegruppen vor Ort aufmerksam und motivieren sie, dort Mitglied zu werden. Dies ist besonders hilfreich, da in vielen Fällen Vorurteile gegenüber der Selbsthilfe dazu führen, dass Hilfesuchende nicht den Weg in die Selbsthilfegruppe finden [10]. Die Unterstützung der Selbsthilfegruppen ist auch für die Schlaganfall-Helfer*innen hilfreich, da die Gruppen nach der Beendigung der Begleitung durch die Helfenden für die Hilfesuchenden eine dauerhafte Anlaufstelle und somit auch für die Helfenden die Option einer sanften Exit-Strategie aus der jeweiligen Begleitung sein kann.

Gerade für ein hochkomplexes Erkrankungsbild wie den Schlaganfall können Schlaganfall-Helfer*innen im Sinne der Rolle eines „Kümmerers“ [6, 13] dazu beitragen, Versorgungslücken in der Langzeitnachsorge zu schließen. Diese Form der Unterstützung kann auch beispielhaft für andere ähnlich komplexe Erkrankungen sein.

## Ausblick

Für die Zukunft wird die Weiterentwicklung des Projektes im Hinblick auf onlinebasierte Schulungsmodule ein weiterer Schritt in Richtung einer flächendeckenden Umsetzung sein. Damit eng verbunden ist die gezielte Ansprache überregionaler Verbände, die als Partnerorganisationen gewonnen werden können.

## Fazit für die Praxis

- Die bisherigen Erfahrungen mit dem Projekt „Geschulte Schlaganfall-Helferinnen und Schlaganfall-Helfer – ein Partnerschaftliches Modell für die wohnortnahe Versorgung (GeSa)“ sind durchgehend positiv. Die Schlaganfall-Helfer*innen werden als „sinnvolle Ergänzung zur Regelversorgung“ beschrieben.
- Als besonderer Vorteil der ehrenamtlichen Schlaganfall-Helfer*innen wird angeführt, dass die persönliche Betroffenheit von ihnen Angehörige und Betroffene zusätzlich motivieren kann.
- Professionelle Leistungserbringer*innen profitieren, da die Helfer*innen ihnen zeitaufwendige Beratungsleistungen abnehmen.
- Die organisatorischen Rahmenbedingungen in Form des Vorhandenseins einer Projektkoordination beim Helfer*innen-Projekt vor Ort wurden als positiver Erfolgsfaktor identifiziert. Insofern kann die bereits im Modellprojekt umgesetzte Einbindung von Kooperationspartner*innen für die Koordination der Ehrenamtlichen als einer der wichtigsten Erfolgsfaktoren für die Nachhaltigkeit des Projekts bezeichnet werden.

## Footnotes

1 Alternativ zur Abrechnung durch den jeweiligen Schlaganfall-Helfer*innen kann auch die Organisation, die das Helfer*innen-Projekt vor Ort betreut, die Abrechnung für alle Helfer*innen-Einsätze durchführen. In beiden Fällen (Abrechnung als Einzelperson oder Abrechnung als Organisation) muss im Vorfeld zusätzlich eine Anerkennung bei der zuständigen Behörde vor Ort erfolgen.

2 Beispielsweise werden vom Schlaganfall-Ring in Schleswig-Holstein ausgebildete Schlaganfall-Helfer*innen auch als „Mentor*innen“ bezeichnet. Für die Helfer*innen-Schulungen mit dem Bayrischen Roten Kreuz wurde ein zusätzliches Schulungsmodul zum Notfallmanagement erarbeitet.

3 Das „Manual zur Organisation einer Schlaganfall-Helfer*innen-Schulung“ enthält ausführliche Beschreibungen zur Gesamtplanung, zur Öffentlichkeitsarbeit, zu Finanzierungsmöglichkeiten, zur Partner*innenakquise, zu den einzelnen Schulungsmodulen und zur Nachbetreuung. Zusätzlich sind weitere Dokumente wie Checklisten und Musterformulare enthalten.

4 Dies ist nicht zwingend erforderlich und auch nur dann möglich, wenn die zeitlichen Ressourcen der Projektkoordination dies überhaupt zulassen.

5 Auch dies ist nur möglich, wenn die zeitlichen Ressourcen der Projektkoordination dies zulassen.

6 Im Erstkontaktbogen können die genaue Form der Unterstützung, ein Ziel und die Dauer der Hilfe fest definiert werden. Außerdem bietet der Bogen Platz für verschiedene andere personenbezogene Daten und weitere Notizen. Insgesamt ist es durch diese Form der Dokumentation möglich, einen Fall auch später nachvollziehen zu können. Dies ist insbesondere dann von Vorteil, wenn andere Schlaganfall-Helfer*innen zu einem anderen Zeitpunkt mit dem Fall zu tun haben.
